# Lessons Learned From a Living Lab on the Broad Adoption of eHealth in Primary Health Care

**DOI:** 10.2196/jmir.9110

**Published:** 2018-03-29

**Authors:** Ilse Catharina Sophia Swinkels, Martine Wilhelmina Johanna Huygens, Tim M Schoenmakers, Wendy Oude Nijeweme-D'Hollosy, Lex van Velsen, Joan Vermeulen, Marian Schoone-Harmsen, Yvonne JFM Jansen, Onno CP van Schayck, Roland Friele, Luc de Witte

**Affiliations:** ^1^ Netherlands Institute for Health Services Research Utrecht Netherlands; ^2^ Centre for Care Technology Research Maastricht Netherlands; ^3^ Department of Health Services Research School for Public Health and Primary Care Maastricht University Maastricht Netherlands; ^4^ Department of Clinical Psychology University of Amsterdam Amsterdam Netherlands; ^5^ Telemedicine Biomedical Signals and Systems University of Twente Enschede Netherlands; ^6^ Roessingh Research & Development Enschede Netherlands; ^7^ Lunet Zorg Eindhoven Netherlands; ^8^ Work Health Technology Expertise Group Netherlands Organisation for Applied Scientific Research TNO Leiden Netherlands; ^9^ Robuust Eindhoven Netherlands; ^10^ Department of Family Medicine School for Public Health and Primary Care Maastricht University Maastricht Netherlands; ^11^ Tranzo Tilburg School of Social and Behavorial Sciences Tilburg University Tilburg Netherlands; ^12^ Research Center Technology and Care Zuyd University of Applied Sciences Heerlen Netherlands

**Keywords:** telemedicine, primary health care, implementation, patient involvement, entrepreneurship, health personnel, policy makers

## Abstract

**Background:**

Electronic health (eHealth) solutions are considered to relieve current and future pressure on the sustainability of primary health care systems. However, evidence of the effectiveness of eHealth in daily practice is missing. Furthermore, eHealth solutions are often not implemented structurally after a pilot phase, even if successful during this phase. Although many studies on barriers and facilitators were published in recent years, eHealth implementation still progresses only slowly. To further unravel the slow implementation process in primary health care and accelerate the implementation of eHealth, a 3-year Living Lab project was set up. In the Living Lab, called eLabEL, patients, health care professionals, small- and medium-sized enterprises (SMEs), and research institutes collaborated to select and integrate fully mature eHealth technologies for implementation in primary health care. Seven primary health care centers, 10 SMEs, and 4 research institutes participated.

**Objective:**

This viewpoint paper aims to show the process of adoption of eHealth in primary care from the perspective of different stakeholders in a qualitative way. We provide a real-world view on how such a process occurs, including successes and failures related to the different perspectives.

**Methods:**

Reflective and process-based notes from all meetings of the project partners, interview data, and data of focus groups were analyzed systematically using four theoretical models to study the adoption of eHealth in primary care.

**Results:**

The results showed that large-scale implementation of eHealth depends on the efforts of and interaction and collaboration among 4 groups of stakeholders: patients, health care professionals, SMEs, and those responsible for health care policy (health care insurers and policy makers). These stakeholders are all acting within their own contexts and with their own values and expectations. We experienced that patients reported expected benefits regarding the use of eHealth for self-management purposes, and health care professionals stressed the potential benefits of eHealth and were interested in using eHealth to distinguish themselves from other care organizations. In addition, eHealth entrepreneurs valued the collaboration among SMEs as they were not big enough to enter the health care market on their own and valued the collaboration with research institutes. Furthermore, health care insurers and policy makers shared the ambition and need for the development and implementation of an integrated eHealth infrastructure.

**Conclusions:**

For optimal and sustainable use of eHealth, patients should be actively involved, primary health care professionals need to be reinforced in their management, entrepreneurs should work closely with health care professionals and patients, and the government needs to focus on new health care models stimulating innovations. Only when all these parties act together, starting in local communities with a small range of eHealth tools, the potential of eHealth will be enforced.

## Introduction

### Needs for Real-World View on eHealth Adoption

Electronic health (eHealth) solutions are expected to empower patients and maintain or improve health outcomes, while generating cost-effective gains and lowering primary health care professionals’ workload [1,2]. However, it appears to be difficult to embed them in daily health care routines [3]. Often, use of eHealth services stops when research projects are finished, even when successful. Moreover, there is still uncertainty about their effectiveness in daily practice [2,4,5]. Therefore, the success rate to date of eHealth in primary health care is low [6]. Current evidence on eHealth and care technologies is mainly based on clinical trials and isolated eHealth applications. Van Gemert-Pijnen et al (2011) suggest that evaluations should not focus exclusively on measuring outcome variables (via randomized controlled trials) but should also include in-depth process data concerning the usage of eHealth [7].

It is suggested that successful implementation of eHealth asks for a complex innovation approach [6]. Numerous factors are related to its success, including characteristics of the end users, the function and usability of the intervention, the technical infrastructure, change management of health care organizations, the health care system, and financial business models [2,4,6,7]. It can be stated that 4 groups of stakeholders are responsible for a successful implementation of eHealth solutions: patients, health care professionals, entrepreneurs, and those responsible for health care policy (policy makers and health care insurers) [8]. Eysenbach (2001) stated in 2001 that eHealth is an emerging field at the intersection of medical informatics, public health, and businesses [9]. However, literature combining the views of these different fields and describing their challenges systematically is scarce.

With this paper, we aim to fill this gap and will describe the challenges that arose when patients, health care professionals, and entrepreneurs collaborated in a Living Lab setting to select, integrate, implement, and evaluate eHealth in primary health care. Hereby, our aim is not to test the relationships and interactions between different factors and stakeholders. Rather, we aim to show, in a qualitative way, the process of adoption of eHealth in primary care from the perspective of different stakeholders. This provides a real-world view on how such a process occurs, including successes and failures related to the different perspectives. Our paper serves as an illustration that underlines the importance of including all 4 stakeholders, having a shared vision statement, and enabling all partners to invest time or money, as only then can the expected potential of eHealth solutions be reached. After providing the rationale for our Living Lab project and a short description of our methods, we reflect upon our findings in 4 sections—patients as stakeholders; health care professionals as stakeholders; entrepreneurs as stakeholders; and health care insurers and policy makers as stakeholders. On the basis of these findings, we have been able to develop lessons learned, which seem to be important in positively shaping the outcome of eHealth implementation and adoption in future primary health care.

### Rationale for the Living Lab Project “eLabEL”

In 2012-2013, when writing the grant proposal for the eLabEL project, it was already known that much of the eHealth technology being developed did not reach primary care practice because of a suboptimal fit between the needs in primary care and the technical solutions [10]. Simultaneously, there was very little knowledge about what it takes to bring such technologies into practice.

With eLabEL, we aimed to contribute to this knowledge, and to bridge technology and implementation. We believed, and still believe, that incorporating eHealth into daily practice is essential for optimal effects on quality and efficiency of health care. In other words, traditional health care should change to “technology-supported health care.” For such a change, not only a technological innovation but also a societal innovation is essential. Furthermore, according to Van Velsen and colleagues (2013), a multidisciplinary development approach is necessary [11]. Van Gemert-Pijnen et al (2011) stated that relevant stakeholders should collaborate, and research should consist of qualitative and quantitative elements [7].

From this perspective, we, as researchers, started the eLabEL project in 2013, together with entrepreneurs, patients, and health care professionals. eLabEL was aimed at establishing a Living Lab in which patients, health care professionals, entrepreneurs, and researchers could collaborate during the selection, integration, implementation, and evaluation of mature eHealth-tools in primary health care [12]. According to the European Network of Living Labs, we defined a Living Lab as a user-centered, open innovation ecosystem based on a systematic user cocreation approach, integrating research and innovation processes in real-life communities and settings [13].

In this project, the focus was on two types of mature eHealth technologies: (1) online communication services which can be used by all patients in the practice and (2) eHealth for self-management purposes for those with a chronic somatic condition. Textbox 1 provides a description of the eLabEL project, and Table 1 provides a description of the characteristics of the participating primary health care centers.

**The eLabEL project as illustration.**

The eLabEL project was conducted from September 2013 until December 2016 in the Netherlands. We aimed at the establishment of Dutch Living Labs in which integrated eHealth applications would become part of regular health care. Concurrently, we aimed to study the consequences of the integration of eHealth applications in primary care, as well as technical barriers and facilitators.

Seven primary health care centers participated in eLabEL. These were recruited via the network of the participating research partners or positively responded to the recruitment call that was published in a press release and at the project’s website. In these centers, at least one general practitioner, physical therapist, practice nurse, and nurse assistant provided health care to the community. Participating practices varied in type of organization, experiences with eHealth, patients’ characteristics, and region. Characteristics of these health care centers can be found in Table 1.

Patients of these primary health care centers were also invited to participate. Ten enterprises participated in the Living Labs. These were mainly small- and medium-sized enterprises (SMEs) and offered different eHealth applications or services, varying from videoconferencing and online coaches for patients with chronic diseases to activity sensors and data warehousing. These SMEs were recruited via the network of the participating research partners. Some of them already participated in prior research projects. Also 4 research institutes, collaborating in the Centre for Care Technology Research, participated. These profit and nonprofit organizations collaborated to select and integrate mature eHealth technologies for implementation in primary care. One or two members of each research institute coordinated the project.

In the Living Lab patients, health care professionals, entrepreneurs, and researchers were invited to have close contact with each other during the whole project. In practice, the following activities took place:

- At the start of the project, needs and expectations of patients and health care professionals were inventoried via focus groups and interviews. These needs were linked to existing eHealth applications developed by the SMEs.
- Regular group sessions were held with the SMEs in which they discussed integrating technology and explored a viable business model.
- Two group sessions were held with health care professionals from all participating centers.
- Two group sessions were held with health care professionals and entrepreneurs.
- Regular meetings were held with the individual practices.
- Meetings were held with policy makers and health care insurers.

The final eLabEL package exists of the following eHealth applications:

- A service to provide online video consultations
- An online self-management coach for people with chronic obstructive pulmonary disease (COPD), which supports them into a healthier lifestyle, monitors their health status, and signals decline of health status
- An online coach for patients under treatment by the physiotherapist to support them in doing exercises at home by giving online training schemes and videos
- An application developed to coordinate multidisciplinary care around a patient. In this application, patients were able to add health care professionals, family, or other caregivers and those persons could read and share information

These applications were integrated in one infrastructure with single sign on for patients.

**Table 1:** Characteristics of the participating primary health care centers. Ca: circa.

Number	Organization	Number of patients (2013)	Region of the Netherlands	Remarks
1	Health care center	Ca 13,000	Mid	
2	Health care center	Ca 8000	South	Located in deprived urban area
3	General practice	Ca 3500	South	
4	Health care center	Ca 5000	West	Located in deprived urban area
5	General practice	Ca 5500	North	Patients mainly students
6	Health care center	Ca 14,500	Mid	
7	General practice	Ca 6500	North	

## Methods

A qualitative design was used to study the processes of adoption of eHealth in our project. We systematically analyzed all reflective and process-based notes from meetings with health care professionals, the scientific project members, members of the management team of the Centre for Care Technology research, members of societal organizations, health care insurers, and enterprises. Furthermore, data from interviews and focus groups on the needs and expectations of health care professionals and patients were included in the analyses, as well as interviews on adoption and implementation of eHealth.

In total, 30 patients with a chronic disease, that is, diabetes, chronic obstructive pulmonary disease (COPD), or a cardiovascular condition, participated in 5 semistructured focus group interviews in the first year of the project. Those patients were recruited in 4 primary care centers by the health care professional. Mean age was 68 years and 73% (22/30) were male. In these focus groups, the following themes were discussed: (1) the impact of the chronic disease on patients’ daily life, (2) their opinions and needs regarding self-management, and (3) their expectations and needs regarding, and willingness to use, eHealth for self-management purposes. See Huygens et al (2016) for a detailed description of the focus group method [14]. In addition, 30 health care professionals (9 general practitioners, 8 physical therapists, 8 nurse practitioners, and 5 supporting staff members from the eLabEL practices) were interviewed in the first months of the project. Themes discussed in these semistructured interviews were (1) the centers’ technical infrastructure, (2) positive and negative work-related experiences with information technology, and (3) future expectations and needs of eHealth. See Oude Nijeweme-d’Hollosy et al (2015) for a detailed description of the interview method [15]. Eight care managers from the eLabEL-practices were interviewed in 2016. In these interviews, the expected facilitating and inhibiting factors for adoption and implementation of the eHealth tools were discussed.

For the analyses, we used a coding scheme based on four theoretical models to initially structure our findings. Wagemakers’ model (2010) focuses on collaboration among multidisciplinary organizations in health care [16]. Nystrom’s model (2014) was used because of its focus on different role approaches within a collaboration [17]. The model of Geels (2002) describes new technologies as arising and maturing within existing technology systems [18]. Fleuren et al (2004) state that the success rate of an innovation is dependent on the level of the innovation itself, end users, organization, and the social-political context [19]. All elements in these models are included in the coding scheme. More information about these theoretical models can be found in Multimedia Appendix 1.

To shed light on the process of implementation, we performed a qualitative summative process evaluation, in which analyses were performed 21 months after the start of the project and at the end of it. At 21 months after the start, documents were allocated among some of the authors (IS, MH, WH, LV, JV, MS, and YJ). Each set of documents was coded using the coding scheme and then thematically summarized by the author. Each summary was then checked by 1 researcher of another research institute. At the end, for pragmatic reasons, 1 researcher (MH) coded and summarized the last set of documents, and 4 researchers checked the summary (IS, WH, LV, and MS). A summary of the findings of both rounds of analyses was shared with the SMEs and health care professionals for their confirmation (member check procedure). Furthermore, YJ has observed the project as action researcher. On the basis of these procedures, the findings were used to describe the process of implementation from the perspectives of the identified stakeholders.

## Results

### Patients

#### Envisaged Role in eLabEL

eLabEL aimed at a user-centred design. Patients were intended to be actively involved in the selection and implementation of eHealth solutions. With actively involved, we mean that their input is collected and used from the start (selection phase) to the end (implementation). This way, we expected to stimulate the use of eHealth by patients. However, active patient involvement was only achieved to a minor extent. In addition, we found that patient involvement does not always guarantee usage of specific eHealth technologies on a broad scale because not every patient seems willing to use eHealth.

#### Patient Involvement in Research

The first way to involve patients was by organizing focus groups to investigate their expectations and needs regarding eHealth. Patients had to be recruited by health care professionals to participate in these group interviews. However, it was difficult for them to encourage patients to participate. According to the health care professionals, one of the main reasons was that patients were tired of participating in research. Therefore, organizing patient involvement was more time-consuming than expected. In addition to the focus group interviews, we attempted to set up a patient panel for the active involvement of patients during the entire project. However, this resulted in only a few positive responses. We were more hesitant to encourage health care professionals to recruit more patients for this panel, as the first study already required significant effort. Furthermore, throughout the project, actively involving patients to incorporate the patient perspective in the project became of secondary importance. The focus of eLabEL shifted toward the development of an integrated eHealth structure and the investigation of barriers for its slow development and implementation. As a result, health care professionals did not offer it to their patients.

#### Willingness to Use eHealth Differs Between Patients

Despite the difficulties in involving patients, we did organize 5 focus groups with patients with a chronic condition. Detailed results from these focus groups about self-management and use of eHealth are published by Huygens et al [14]. Briefly, it showed that patients reported expected benefits regarding the use of eHealth for self-management purposes. For example, a patient with diabetes reported:

If you can monitor automatically, you get customised care more quickly. Currently, you’re going to the care practice 4 times a year, and in the period in between you stay at the same value [of insulin], while you maybe should have changed it in the meantime, but you didn’t know that.

Focus groups, patient with diabetes

However, many patients also did not feel a need to use eHealth for self-management purposes. It seemed that the perceived benefits of using eHealth should outweigh the negative consequences of frequently having to take action to deal with the disease, which reminds patients about having a disease. A patient with a cardiovascular condition that had little impact on his daily life mentioned the following:

The disadvantage is that I’m feeling more like a patient [because of frequent monitoring]: man suffers most from the suffering he fears.

Focus groups, patient with a cardiovascular condition

### Health Care Professionals

#### Envisaged Role in eLabEL

The role of the health care professionals was to actively participate in the Living Lab settings. They were expected to provide input regarding their own needs and requirements regarding eHealth and its implementation. In addition, they had to use the applications in their daily care processes and encourage and support their patients to use them. Our intention was that health care professionals would implement and use eHealth without the help of the research team. However, we found that the organization of primary health care was inadequate and not sufficiently equipped for doing so as we explain in the upcoming section.

#### Health Care Professionals See Potential in eHealth

The participating health care professionals stressed the potential benefits of eHealth. Professionals identified the rising development of eHealth technologies, the emergence of different eHealth initiatives, and their opportunities for better health care. In addition, care professionals indicated that they were interested in using eHealth to distinguish themselves from other care organizations. Providing extramural care, monitoring patients at a distance, empowering and supporting self-management of patients, providing more intensive care in less time, providing care during out-of-office hours, and increasing the quality of care, were frequently mentioned anticipated benefits of eHealth. Health care professionals believed that by using eHealth for people with mild conditions, they could save time and provide extra time to those with more severe conditions.

#### Support for Incorporating eHealth in Daily Practice

After deciding which eHealth technologies they wanted to use, it was not just a matter of connecting the technology. We experienced many difficulties in the implementation of eHealth in the care practices. First, health care professionals needed support for eHealth usage, including clear instruction material, a helpdesk, and, most importantly, time to gain experience with eHealth, as they had not worked with the selected eHealth applications previously. In addition, for health care professionals it was unclear how eHealth could be successfully integrated into their daily work. Workflow, responsibilities, and roles needed to change, and they did not know how to approach this. Moreover, eHealth was not integrated into the electronic medical records or protocols. This made it difficult for the health care professionals to imagine how to integrate eHealth into their daily care processes. Furthermore, health care professionals expected and experienced problems regarding motivating patients to use eHealth. Clear instruction material and tips (eg, from other care professionals) to encourage and convince patients to use eHealth were needed. In addition, health care professionals indicated that they did not want to innovate without the help and encouragement of other health care professionals within and outside their own organization. It appeared that the innovation should fit with the ambitions and plans of the local care community.

#### Convincing Partners Within and Outside the Practice

So, health care professionals needed support on different levels during the implementation, more than we expected. For these support activities funding was needed, which was not covered by the budget for the project. Several care practices tried to apply for eHealth funding. However, we experienced that it was complex for them to organize this. Often, they lacked knowledge, expertise, or resources to apply for eHealth funding. Professionals mentioned that in the current financial model, they had to pay the costs (time and money) for eHealth implementation, while the health insurer would receive the proposed benefits in terms of cost reductions (also known as the wrong pocket problem).

In addition, health care professionals already experienced a high time pressure in regular care processes and in keeping up with bureaucratic and legal changes, resulting in a lack of time to adopt eHealth. Moreover, in most practices, eHealth was not mentioned in vision and mission statements. Furthermore, the care professionals and managers who agreed to participate in the eLabEL project were not the ones that actually had to work with the applications in real practice. An “eHealth-minded” care manager does not guarantee the actual use of eHealth by his or her colleagues when there is no clear vision on eHealth in the care organization or space for innovation. The aforementioned reasons resulted in low priority for eHealth implementation. As summarized by one of the managers:

I am supporter of such innovations in health care, but I also see that they conflict with every day practice. General practitioners are up to their ears in work. They have no time for implementation. Primary health care professionals experience extreme pressure due to the substitution from secondary to primary care, which is bothering them. Besides, it is still unknown what the purpose and target population of eHealth is and why we would use it. That is scary. Then, you can imagine why eHealth has low priority.

Interview, manager primary health care center, March 9, 2016

### Entrepreneurs

#### Envisaged Role in eLabEL

The entrepreneurs’ role in eLabEL was to bring in mature and evidence-based eHealth applications, in conjunction with patients and health care professionals, and to combine the different applications into one infrastructure via a single sign-on. To realize a sustainable, intelligent, and interoperable information and communication technology (ICT) infrastructure, which was necessary for eLabELs’ mission, the individual applications as well as the infrastructure should meet the national and European requirements for data exchange, data safety, and data privacy. The entrepreneurs were also asked for knowledge and financial investments.

#### Small- and Medium-Sized Enterprises Incentive to Collaborate

Participating entrepreneurs started in eLabEL with the expectation that collaboration with research institutes would help them to enter a new market, that is, primary health care. They felt they needed to collaborate with other entrepreneurs as they were not big enough to enter the market on their own. They expected collaboration with research institutes as an important surplus value: it would add a scientific basis for their applications and therefore could create additional market value. They valued the intensive collaboration among the SMEs resulting in small alliances of 2 or 3 SMEs, as well as the experiences of participating in the project as a whole.

#### Small- and Medium-Sized Enterprises Need a Positive Business Case

During the project, it came to the fore that entrepreneurs did not have the technical knowledge that was needed to set up a sustainable interoperable ICT infrastructure and that their eHealth applications were neither fully mature nor evidence based. Furthermore, the entrepreneurs were continuously considering whether investments in eLabEL would result in future revenues (mainly in the short term). As the SMEs differed in their motivation and in weighing investments, it proved hard to create a shared vision statement on the integration of the different eHealth applications, the investment strategy, and a joined entity to assign intellectual properties to. The main reason for the struggles experienced in the cooperation among SMEs was that the SMEs differed in their convictions of future revenues because of uncertainties in the financial market and that it was not possible to make a positive business case. An individual investment in the eHealth infrastructure was considered as unwise and too risky by each SME, and therefore, they opted for a joint investment. However, the business case and corresponding business model should still be positive. Questions like who will pay, who is the customer, and who is the user were difficult to answer during the whole project, as the primary health care market was a new and therefore relatively unknown market for the SMEs. This resulted in continuous discussions on the business model. One of the SME’s explained it as:

The health care market is unknown. Who should pay for it? How can we sell it? The Business Model is unclear. For medical care the health care insurance should pay. For non-medical care a patient or health care organization should pay. This is difficult in primary health care.

Meeting entrepreneurs, September 16, 2014

### Health Care Insurers and Policy Makers

#### Envisaged Role in eLabEL

In the eLabEL project, the expected role of policy makers and health care insurers was that of enabling the health care professionals in experimenting with the use of eHealth in primary care. More precisely, we expected that health care insurers would provide financial support for the appointment of practice nurses.

#### Shared Ambition

Several discussions were held with health care insurers and policy makers. Time after time it was clear that we had a shared ambition: health care insurers and policy makers agreed that it was necessary to work on an integrated infrastructure for eHealth applications to transform traditional primary health care into technology-supported health care. In their view, the Dutch financial legislation offers prospects for financing eHealth applications as there are policy rules, conditions for reimbursement, and incentives for innovation.

#### Cost-Effectiveness Studies Are Needed

Simultaneously, health care insurers were reserved. They needed a business case and insight into cost-effectiveness of the infrastructure that we were developing before they would think about reimbursement or investments. We could not achieve this in the project and therefore, they did not want to support the project. It seemed that health care insurers were mostly interested in short-term effects. In actual practice, the Dutch regulations and legislation seemed to act inconsistently: they argue to stimulate eHealth on one side but require cost-effectiveness studies first on the other side. However, to carry out cost-effectiveness studies in real practice, implementation of eHealth needs legislation and financial regulations first.

Instead of investments by health care insurance, health care organizations themselves might be able to invest in eHealth-applications. However, in the Dutch pay-for-performance-based health care system, the use of eHealth applications that lower the number of consults will also lower the health care professionals’ revenues. Actually, investments by the health care organizations will lead to lower costs for the health care insurers but also lower income for the health care practices. This was explained by one of the managers as follows:

eLabEL is aimed at more efficient care and better quality of care, with the ideal result that patients are more satisfied. But, this should not result in cost savings only for the health care insurance sector...The shared savings principle might be worthwhile.

Meeting entrepreneurs and primary health care managers, November 6, 2014

## Discussion

About 4 years after starting the eLabEL project, we conclude that, despite the hard work and collaboration of many stakeholders, it was not possible to implement eHealth in these Living Labs at this moment in time. One might say that the eLabEL consortium failed in its ambition. However, we gathered in-depth information about the complexity of innovations in primary health care that can help many researchers, entrepreneurs, and policy makers in setting up the next initiatives on this topic. Our experiences in eLabEL taught us that successful use of eHealth needs more than enthusiastic partners. Successful use also depends on the efforts of all stakeholders, their willingness to invest time or money, and shared vision statements. Although it is not easy because of different contexts, values, and expectations, based on the experiences inside and outside eLabEL, we still believe that collaboration between all 4 groups of stakeholders, that is, patients, health care professionals, entrepreneurs, and health care insurers or policy makers is essential. Moreover, we argue that policy, especially the health care insurance market, should be added as a field to Eysenbach’s definition of eHealth [9].

Were we naive when starting eLabEL? We might be: we knew we were ambitious, but looking back, we realize we had unrealistic expectations and our goals were not specific enough. Nevertheless, SMEs were willing to collaborate and to invest as they were ready to step into a new market. Additionally, health care organizations also felt the urge to participate. What we did not foresee was the struggle (1) to convince health care insurers to support health care professionals in our project and (2) to create a positive business model. In fact, it was those factors that led to an impasse: without commitment of health care centers or insurers, no positive business model could be created, and SMEs could not invest in the eLabEL infrastructure. However, without investments in the eLabEL infrastructure, health care professionals were not convinced of its added value. Moreover, without financial support by health care insurers, they were not motivated or able to use it. This made it impossible for the researchers to collect the evidence that health care insurers were asking for.

With this paper, we aimed to show the process of adoption of eHealth tools in primary care illustrated by the eLabEL project, which provided us a rich qualitative dataset. However, our study has some limitations. The main limitation is the involvement of patients, health care insurers, and policy makers. Patients were supposed to be part of the Living Lab. They were actively involved at the start of the project. Slow progress in development and implementation changed the focus of the project and resulted in less involvement of patients. Health care insurers and policy makers were not part of the Living Lab and were therefore less involved in the project. Nevertheless, it is clear from our observations that a number of actions should be done differently in future projects to enforce the implementation of eHealth in primary care. These actions will be discussed in the following sections and are listed in Textbox 2.

It turned out that it is not easy to actively involve patients in research projects with an eHealth-topic. Considering the importance of their participation, especially in eHealth projects [20], they should be supported in participating in the project. Wildevuur et al (2017) recently published 4 preconditions for enhancing the partnership in ICT-enabled person-centered care [21]. In addition, incentives for care practices seem to be needed to recruit patients for participation. Patient’s expectations of the benefits of using eHealth play an essential role in their actual use. Therefore, it is essential that Living Labs as set up in eLabEL awake patients’ interest by offering relevant eHealth tools. Patients’ expectations are not only dependent on the technology but also on the way in which general practices offer, promote, and use it [22,23]. Care professionals should be supported in informing patients about the possibilities, uses, and reasons for implementation, focusing on the benefits eHealth can bring. However, whether patients will actually use eHealth will always be personal and differ among patients. Monitoring which patients benefit the most from the use of eHealth and those who do not, seems to be important to develop optimal implementation strategies.

From the care organizations’ perspective, it appeared that health care managers and professionals were not ready to implement eHealth tools without support. Implementing eHealth requires it to be a fundamental part of the mission and vision of the health care organization. Only then decisions on budget and support can be made. We found that the process of adapting and implementing eHealth is too complicated to organize next to regular care-giving activities for primary health care professionals. In addition, we learned that involving a primary health care center in the plans is not sufficient.

**Lessons learned from a Living Lab on broad adoption of electronic health (eHealth) in primary health care.**

- Patients need support to actively participate in eHealth projects, and those projects need to be relevant for the patients
- Incentives for care practices are needed to recruit patients for participation in eHealth projects
- Primary care practices need support to adequately inform patients and monitor which patients benefit from the use of eHealth
- The community in which a primary health care system operates needs to be involved in eHealth projects
- Primary care practices need support and managerial power for the implementation and innovation processes
- Collaborated eHealth entrepreneurs need trust in each other, shared vision statements, and early commitment to short- and long-term goals
- A business model concept is needed early in eHealth projects and essential for collaboration
- Strategies are needed focusing on financial models that stimulate innovation and on requirements needed for societal innovations
- Patients, primary health care professionals, entrepreneurs, and government need to act together in eHealth projects

The use of eHealth goes beyond the own practice borders as primary health care professionals often operate close to other health care professionals in their region. This makes the innovations even more complex as those parties also need to be involved [24]. Nowadays, it is common knowledge that implementation of innovations, including eHealth, is difficult and progresses only slowly [25-27]. Lau et al (2016) stated in a recent systematic review of reviews that *implementing any type of change in primary care is likely to be complex* and that *relevant barriers and facilitators are dynamic and likely to change over time* [25]. Theoretical models show that the innovation process or implementation infrastructure are important parts of implementation, next to the intervention characteristics, the organizational structure, the context, and the individuals [8,28]. Moreover, it is shown that a greater knowledge of essential adjustments in health care provider workflow, roles, and responsibilities is needed [29]. Our study provides a real-world view on these topics showing that Dutch primary care organizations, mainly small organizations, do not have the managerial power that is needed for complex innovations such as large-scale eHealth implementation. Primary care organizations probably will benefit from infrastructure that support them in the implementation process.

From the entrepreneurs’ perspective, it is important to have shared vision statements and a business model concept as early as possible. Clearly defined short- and long-term goals are needed. In addition, trust in each other and commitment of all parties is important. Knowledge of the potential cost-effectiveness of eHealth is an important requirement for all stakeholders. The use of an early health technology assessment can provide insight into potential outcomes, drivers, and barriers. Moreover, we should realize that SMEs might have difficulties, due to lack of knowledge, in developing interoperable eHealth, when facing different Dutch and European requirements on data safety, data exchange, and data privacy. Furthermore, in developing, adapting, selecting, and implementing eHealth tools, they should work as closely as possible with the end user, that is, health care professionals and patients. Active user involvement is a time-consuming process. Developers should balance the need for input from users, with the availability of resources such as time and funding [30]. To remain competitive within a fast-moving market, it is important to develop quickly [31]. However, we recommend that the need assessment phase should not be neglected; this seems of major importance for the development of eHealth from which patients can experience benefits and might be an important trigger to actually use eHealth.

The implementation of eHealth is not yet a fully recognized aspect of primary health care organizations, which makes it difficult to fit eHealth locally. Furthermore, inconsistencies in policy rules hinder improvements and innovations. For projects such as eLabEL, it would help when policy makers and health care insurers would allow experiments in which standard regulations can be (partly) neglected to fully explore new financing models. This can only be arranged when policy makers and health care insurers are involved from the beginning of the project. However, such experiments are not a structural solution for broad-scale implementation of eHealth. Moreover, financial support does not guarantee the large-scale use of eHealth [32]. Broad-scale implementation will need strategies that not only focus on financial models that stimulate innovation but also on requirements needed for societal innovations [33,34].

In conclusion, we believe that for optimal and sustainable use of eHealth, patients should be actively involved, primary health care professionals need to be encouraged in their management, entrepreneurs should work closely with health care professionals and patients, and government needs to focus on new health care models stimulating innovations. Only when all these parties act together, starting in local communities with a small range of eHealth tools, the potential of eHealth will be realized.

## Appendix

Multimedia Appendix 1 Theoretical models.

## Abbreviations

COPD: chronic obstructive pulmonary disease
eHealth: electronic health
ICT: information and communication technology
SMEs: small- and medium-sized enterprises
