# Supplementary material for: Lessons Learned From a Living Lab on the Broad Adoption of eHealth in Primary Health Care
Source: J Med Internet Res. 2018 Mar 29;20(3):e83. doi: 10.2196/jmir.9110 (PMC5897624; doi:10.2196/jmir.9110)
Supplement: Multimedia Appendix 1 [file jmir_v20i3e83_app1.pdf]

Multimedia Appendix 1: Theoretical models

An extensive and systematically analysis was performed based on all documentation which was written and collected during the first 21 months of the project, including all notes of meetings, documentation of bilateral sessions, and progress reports, as well as reports of the interviews and focus groups with respectively healthcare professionals and patients. We used four theoretical models to structure the data:

1. Wagemakers et al. (2010) states that collaboration among multidisciplinary organizations, like science, business and healthcare, is important and enlarges the chances on successful innovations in healthcare. Cooperation leads to a socially desirable climate which stimulates uptake of innovations in several levels [15]. This model structures the way how the different partners in eLabEL collaborated.
2. Nyström et al. (2014) states that innovations are increasingly the result of cooperation between organizations and of network structures including public-private-people-partnerships. The success of such partnerships depends on the way how partners collaborate, which roles they take and which roles they obtain by others [16]. They define four categories to describe different role approaches within a collaboration:
    a. Roles that are predetermined by the actors in the network.
    b. Roles that are created in a social structure, such as a network.
    c. Roles that are used as a resource to control resources or establish structure.
    d. Roles that are determined by actions and based on openness and common goals of the network.

   We used these roles to describe the relations between the partners.
3. Geels (2002) states that new technologies arise and mature within existing technology systems. Factors like financial incentives, strategies of consortium partners, competition, and new insights influence (the success of) innovations [17]. Geels' model was used to describe eLabEL as innovation within an existing technological system.
4. Fleuren et al. (2004) assumes that innovations have various determinants and that its' implementation has various phases [18]. The success rate of an innovation is dependent on the level of the innovation itself, end-user, organization, and social-political context.

The research group combined the elements of these four models in a coding scheme. The coding scheme was tested in a subset of the documents. All researchers (n=6) coded this subset. The coded documents were discussed by the research group (n=6) to ensure all researchers applied the scheme in the same way. The research group discussed the findings and minimally adapted the scheme. Subsequently, the researchers performed content analyses of the documents by using this coding scheme. Documents were thematically allocated among the members. Each set of documents was coded and then summarized by one researcher. Each summary was then checked by a researcher of another research institute.
